# Transposable elements as genome regulators in normal and malignant haematopoiesis

**DOI:** 10.1038/s41408-025-01295-9

**Published:** 2025-05-06

**Authors:** Dmitry Prokopov, Hale Tunbak, Eve Leddy, Bryce Drylie, Francesco Camera, Özgen Deniz

**Affiliations:** 1 Centre for Haemato-Oncology, Barts Cancer Institute, Queen Mary University of London, London, UK; 2 QMUL Centre for Epigenetics, Queen Mary University of London, London, UK

**Keywords:** Haematological cancer, Haematopoietic stem cells, Cancer genomics

## Abstract

Transposable elements (TEs) constitute over half of the human genome and have played a profound role in genome evolution. While most TEs have lost the ability to transpose, many retain functional elements that serve as drivers of genome innovation, including the emergence of novel genes and regulatory elements. Recent advances in experimental and bioinformatic methods have provided new insights into their roles in human biology, both in health and disease. In this review, we discuss the multifaceted roles of TEs in haematopoiesis, highlighting their contributions to both normal and pathological contexts. TEs influence gene regulation by reshaping gene-regulatory networks, modulating transcriptional activity, and creating novel regulatory elements. These activities play key roles in maintaining normal haematopoietic processes and supporting cellular regeneration. However, in haematological malignancies, TE reactivation can disrupt genomic integrity, induce structural variations, and dysregulate transcriptional programmes, thereby driving oncogenesis. By examining the impact of TE activity on genome regulation and variation, we highlight their pivotal roles in both normal haematopoietic processes and haematological cancers.

## Introduction

**Fig. 1:**
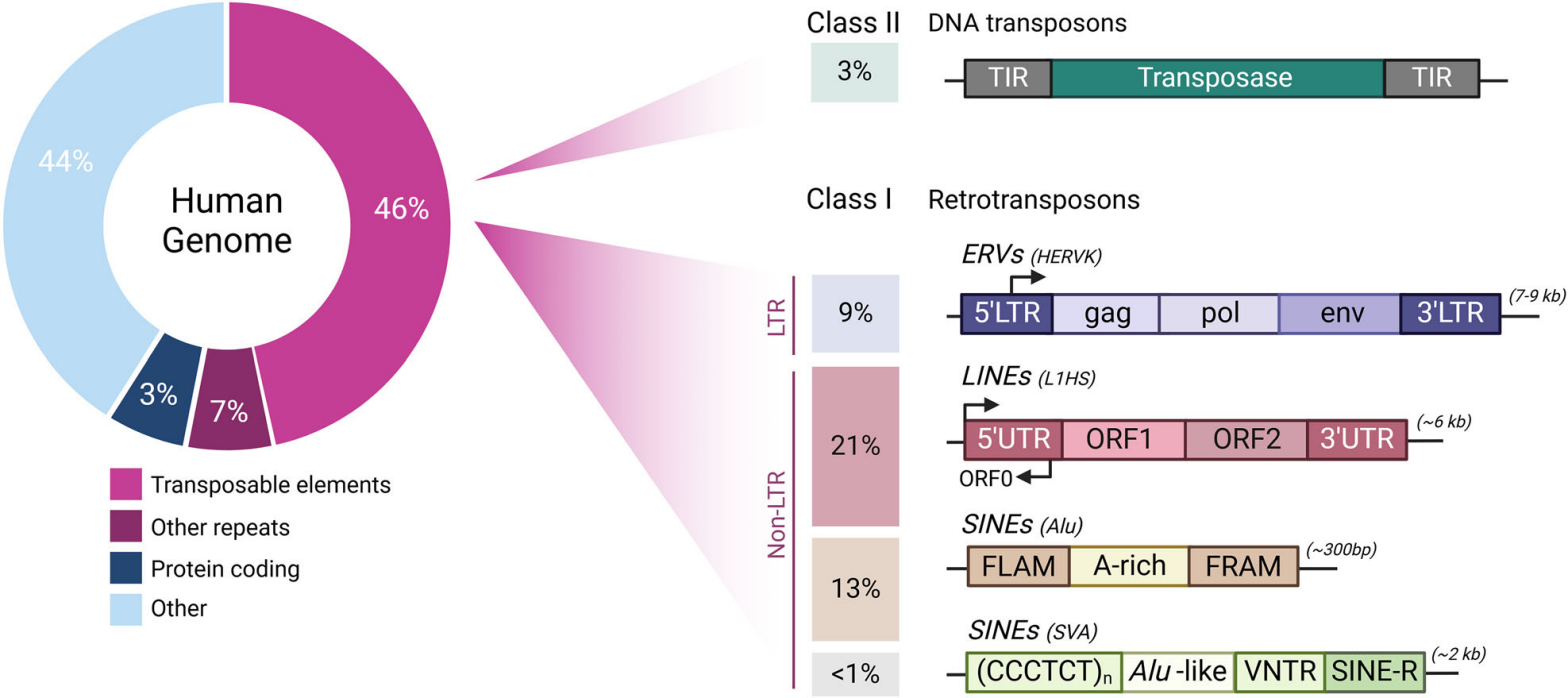
Transposable elements in the human genome. TEs occupy 46% of the human genome based on the the telomere-to-telomere (T2T) CHM13 reference genome annotation (left). TEs are divided into two major classes based on their transposition mechanism- Class I TEs (retrotransposons) use copy-and-paste mechanism and are further subdivided into long terminal repeat (LTR), long interspersed nuclear element (LINE) and short interspersed nuclear element (SINE) superfamilies. LINE-1s are the only autonomous transposons currently active in the human genome. Class II TEs (DNA transposons) use cut-and-paste mechanism to integrate into the genome, however they have lost their ability for transposition in the human genome (right). Created in BioRender.

Transposable elements (TEs) are dispersed repetitive DNA sequences that have the ability to move within the host genome. First discovered in the 1940s by Barbara McClintock in the maize genome [1], TEs are now recognised across all organisms with varying composition and abundance. In humans, over 46% of the genome consists of TEs according to the telomere-to-telomere (T2T) CHM13 reference genome annotation (Fig. 1) [2]. Based on their mode of transposition, TEs are classified into two major classes [3]. Class I TEs (retrotransposons) transpose into the genome via reverse-transcribed RNA intermediates using a “copy-and-paste” mechanism. Retrotransposons are further subdivided into long terminal repeat (LTR) and non-LTR retrotransposons. LTR retrotransposons, including endogenous retroviruses (ERVs), are remnants of ancient integration events [4] and comprise around 9% of the human genome. Full-length ERVs harbour internal coding regions for viral proteins (gag, pol and env), flanked by two LTRs. However, 90% of ERVs exist as solo LTRs in the human genome [5]. Another major subclass of retrotransposons is long interspersed nuclear elements (LINEs). LINEs constitute ~21% of total DNA with ~500,000 copies [6]. Despite their abundance, only about 100 of these copies per genome remain fully functional and capable of retrotransposition [7, 8]. LINE-1 is the only autonomous non-LTR retrotransposon in the human genome, facilitating the mobilization of non-autonomous short interspersed nuclear elements (SINEs). SINEs, including the primate specific Alu elements, comprise around 13% of our DNA. While LINE-1s rely on RNA Polymerase II (Pol II) for their transcription [9], Alu elements encode a weak internal RNA Pol III promoter [10]. Class II TEs are DNA transposons that use a “cut-and-paste” mechanism for transposition and comprise ~3% of the human genome. There are no endogenous DNA transposons that are capable of transposition in the human genome [11].

Although the vast majority of TEs in humans have lost their ability to retrotranspose due to accumulated mutations or host-mediated repression, many still retain functional sequences that have significantly impacted genome evolution [12]. This includes the domestication of TEs, a process by which originally mobile genetic elements are co-opted for new functions within the host genome. Notable examples of domesticated TEs include DNA transposon-derived piggyBac transposable element-derived protein 5 (PGBD5) [13], which induces site-specific DNA rearrangements in human cancers [14]; the recombination-activating genes *RAG1* and *RAG2*, derived from Transib DNA transposons, which play a central role in the V(D)J recombination process [15]; and ERV-W-derived *Syncytin* gene, which contributes to placental development in mammals [16].

**Fig. 2:**
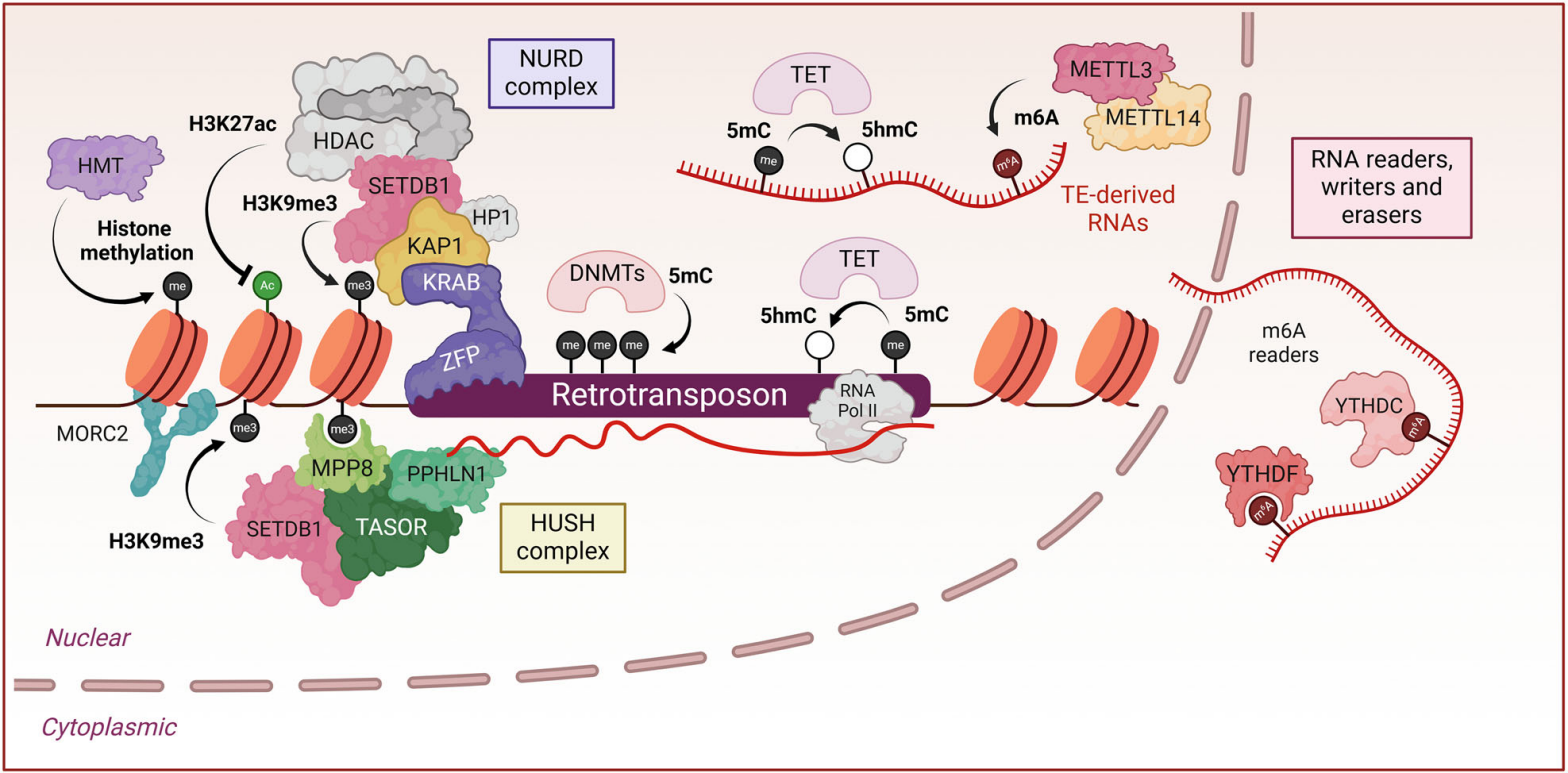
Regulatory mechanisms to control transposable elements. Various transcriptional and post-transcriptional mechanisms control the activity of TEs. Epigenetic repression of TEs is primarily achieved through the sequence-specific recognition of TE elements by KRAB-Zinc Finger Proteins (KRAB-ZFPs). These proteins recruit the KAP1 cofactor, which, in turn, facilitates the formation of a repressive chromatin structure by recruiting histone modifying enzymes, such as SETDB1 and histone deacetylase (HDAC), leading to histone methylation and deacetylation. DNA methyltransferases (DNMTs) deposit repressive DNA cytosine methylation marks on TE sequences, further silencing their activity. The maintenance of this silencing is supported by DNA methylation maintenance mechanisms during replication, primarily involving DNMT1 and UHRF1. Additionally, the human silencing hub (HUSH) complex targets young TE families and recruits SETDB1 to silence target TEs. Moreover, RNA modifications, including m^6^A and m^5^C, influence TE transcript stability, splicing, and translation, adding an additional layer of regulation. Created in BioRender.

Beyond domesticated elements, other functional TEs continue to influence genome regulation, contributing to both genetic and epigenetic diversity with far-reaching consequences for both normal cellular processes and disease states. Derepression of TEs in cancer, for example, can lead to aberrant oncogene expression, inactivation of tumour suppressor genes or trigger genomic instability. Conversely, TE activation can contribute to cellular plasticity and shape lineage-specific transcriptome during normal haematopoiesis. In this review, we first provide an overview of the regulatory mechanisms that modulate TE activity (Box 1; Fig. 2). We then examine the multifaceted roles of TEs in shaping normal and pathological haematopoietic processes, focusing on their gene regulatory activities and contribution to structural genomic variations.

Box 1 Regulatory mechanisms to modulate TE activity TEs are tightly regulated by several interconnected transcriptional and post-transcriptional mechanisms during early development and throughout the entire human life. One of the key regulatory mechanisms to silence TEs is mediated by the Krüppel-associated box domain-containing zinc finger protein (KRAB-ZFP) family, the largest family of transcription factors (TFs) in mammalian cells comprising 378 members in the human genome [120]. KRAB-ZFPs recognise their TE targets in a DNA sequence-specific manner [121] and exert their repressive activity through recruitment of KRAB-associated protein 1 (KAP1), which acts as a scaffold protein for heterochromatin inducers, including SET domain bifurcated 1 (SETDB1), the nucleosome remodelling and deacetylase (NuRD) complex, heterochromatin protein 1 (HP1) and DNA methyltransferases (DNMTs) [122]. Notably, KRAB-ZFPs are thought to have co-evolved with TEs, participating in an evolutionary-arms race and diversifying as new TE families emerge, helping to regulate and suppress their activities [123, 124]. However, evolutionarily younger TEs have evaded KRAB-ZFP repression through the loss of binding sites [125, 126] and are instead controlled by the human silencing hub (HUSH) complex [127–129]. The HUSH complex is an epigenetic silencing complex comprising of three core member proteins; MPHOSPH8 (known as MPP8), FAM208A (referred to as TASOR) and Periphilin-1 (PPHLN1) [130]. Upon target recognition, HUSH recruits Microrchidia CW-type zinc-finger 2 (MORC2) and SETDB1, alongside other tertiary chromatin modifiers to deposit H3K9me3 and establish a heterochromatic environment at target sites [127, 131]. Interestingly, HUSH also regulates some KRAB-ZFPs by recognizing their long terminal 3’ exon ends [128, 130], creating a complex interplay across various TE silencing mechanisms.

DNA methylation, particularly 5-methylcytosine (m^5^C), is a widespread mechanism of TE silencing used by higher eukaryotes [132]. m^5^C involves covalent transfer of a methyl group to a cytosine base neighbouring a guanine (CpG dinucleotide) and is involved in other TE repressive mechanisms, as mentioned above. The evolution of DNA methylation is thought to have been primarily driven by the necessity of silencing TEs which was later co-opted for other functions [133]. Evidence supporting the role of DNA methylation in TE silencing, where the loss of DNA methylation leads to TE activation, has been demonstrated throughout early embryonic development (such as zygotic genome activation and during spermatogenesis), as well as in various cancers, as extensively reviewed here [132].

Small RNAs, including PIWI-interacting RNAs (piRNAs), are another mechanism for TE silencing which function by depositing repressive DNA and histone methylation or degrading TE transcripts [134, 135]. These regulatory mechanisms are predominantly active in the germline, safeguarding genome integrity [136]. In addition, RNA modifications, particularly N6-methyladenosine (m^6^A), play crucial roles in regulating TE activities [137]. m^6^A modifications are catalysed by the complex of methyltransferase-like METTL3–METTL14 enzymes and recognised by various YTH domain reader proteins [137]. m^6^A RNA methylation promotes degradation of transcripts from young LTR subfamilies in mouse embryonic stem cells (ESCs) through their association with YTHDF family readers [138]. Moreover, m^6^A can also mediate TE silencing through the recruitment of SETDB1 [139, 140]. In human cancer cells and ESCs, m^6^A modification, however, positively regulates LINE-1 expression and mobility through the recruitment of specific RNA-binding proteins and cytoplasmic effector eukaryotic initiation factor 3 (eIF3) [141, 142]. Interestingly, the m^6^A reader YTHDC2 can interact with the ten-eleven translocation (TET) enzyme to remove repressive DNA methylation, thereby preventing the epigenetic silencing of ERV elements in human ESCs [143]. These recent findings highlight the complex crosstalk between m^6^A and other epigenetic modifiers in TE regulation in a context and family-dependent manner. Finally, a recent study uncovered an unexpected role for TET2 in oxidising m^5^C on TE RNA, leading to a closed chromatin structure and transcriptional repression [144]. RNA m^5^C modulates chromatin accessibility through interactions with the methyl-CpG-binding protein MBD6, guiding the deubiquitination of the repressive H2AK119ub mark at target TEs. Modulation of TET2 activity in mouse ESCs and in acute myeloid leukaemia (AML) cells reduces this repressive mark, reactivating young retrotransposons through increased m^5^C RNA levels.

## Gene regulatory activity of TEs

**Fig. 3:**
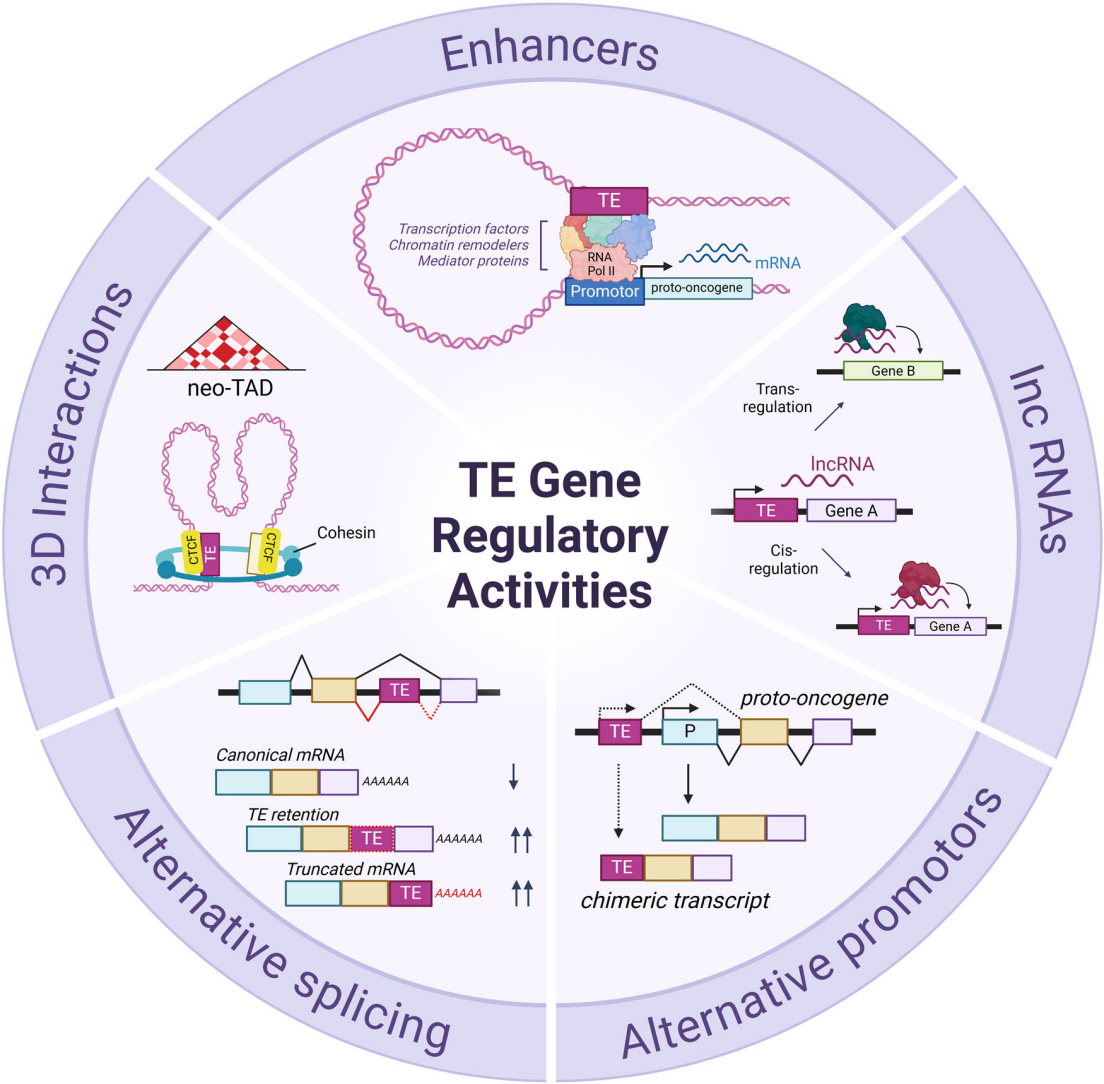
Gene regulatory activities of transposable elements. TEs provide a rich source of *cis*-regulatory elements that are spread across the genome. When active or accessible, the gene regulatory potential of TEs is exploited by the host genome in many ways in normal and malignant haematopoiesis. (i) TEs can act as enhancers through the recruitment of TFs and interacting with nearby promoters, modulating expression. Where this is a proto-oncogene, TEs can contribute to the malignant phenotypes. (ii) TEs can also produce lncRNAs, where 83% of lncRNAs are reported to contain a TE. lncRNAs can have diverse functions from stabilising promoter-enhancer interactions to the recruitment of epigenetic modifiers to chromatin. (iii) TEs can also function as alternative promoters, leading to generation of chimeric transcripts, which may promote a pathogenic phenotype. (iv) Alternative splicing events can occur from the exonisation of TEs, where they may provide alternate splice donor and acceptor sites. These aberrantly spliced transcripts may impair normal protein function, result in mRNA degradation or be translated into neoantigens. (v) TEs are a reservoir for CTCF binding sites and other anchor proteins, thereby retaining the potential to form neo-TADs which can bring *cis*-regulatory sequences to non-target genes and modulate their expression. This can be in the form of enhancer-promoter interactions to increase gene expression, or the inverse insulator-promoter interactions that decrease gene expression. Created in BioRender.

The concept of TEs as influential components of gene regulators dates back to Barbara McClintock’s discovery of their “controlling elements” in maize, where she proposed their role in modulating gene activity [17]. This early insight was further advanced by Britten and Davidson, who suggested that TEs could introduce new regulatory sequences that drive the evolution of gene networks by spreading throughout the genome [18]. Today, we understand that TEs contribute significantly to the regulatory landscape of multicellular organisms, with recent studies from the ENCODE and Roadmap projects indicating that ~25% of candidate *cis*-regulatory elements in the human genome are derived from TEs [19, 20]. TEs contain their own *cis*-regulatory elements and transcription factor (TF) binding sites, which are essential for their transcription and transposition within the genome [20**–**23]. Over evolutionary time, TEs can accumulate mutations, which eventually immobilizes them [24]. However, during this evolutionary process, stochastic mutations (such as CpG deamination) can randomly retain or introduce TF binding sites in TEs. Some of these changes may be preserved if they confer regulatory advantages and are favoured by natural selection, thereby allowing TEs to contribute to tissue- and lineage-specific regulatory networks [25, 26], underscoring their adaptive potential in shaping species- and cell-type-specific landscapes. In this section, we cover examples of TE-derived *cis*-regulatory elements and transcripts in the context of both normal and pathological haematopoiesis (Fig. 3).

### TE-derived alternative promoters

TEs can be integrated into various genomic regions. By introducing their own promoter elements, TEs can create alternative transcription start sites (TSSs) [27], thereby expanding the regulatory potential to neighbouring genes. In fact, it is estimated that between 18–31% of human TSSs overlap with TEs, emphasising their role as promoters in the human genome [28, 29]. Transcription initiated from TE promoters may extend into adjacent genes, generating chimeric transcripts that combine TE sequences with gene exons, enhancing transcriptomic diversity. For example, in murine erythroid cells, loss of transcriptional repressor KLF3 activates an ORR1A0 LTR that drives a chimeric PU.1 transcript with dominant negative activity, promoting erythroid differentiation [30].

In cancer, epigenetic dysregulation often reactivates TE-derived promoters, leading to widespread TE expression. Recent studies have highlighted specific TE expression patterns that correlate with disease subtypes and patient outcomes in haematological cancers, including AML and lymphoid malignancies, although the mechanisms underlying these associations remain unclear [31**–**34].

In some cases, derepressed TEs drive the formation of cancer-specific chimeric transcripts leading to oncogene activation. An example of such a case was reported in Hodgkin’s Lymphoma, where the THE1B retrotransposon acts as an alternative promoter for the colony-stimulating factor 1 receptor (CSF1R) proto-oncogene. Transcription in these cells initiates from a THE1B LTR located ~6.2 kb upstream of the normal TSS. This ectopic expression produces a noncanonical transcript with an extended 5′ UTR, which is essential for tumour cell proliferation and survival [35]. Similarly, the LOR1a retrotransposon activates interferon regulatory factor 5 (IRF5) transcription in the same lymphoma type, promoting overexpression of this factor and supporting cancer cell proliferation [36]. In diffuse large B-cell lymphoma (DLBCL), a chimeric transcript initiated by the activation of an LTR2 element leads to the expression of the modified form of Fatty Acid-Binding Protein 7 (FABP7) [37]. This LTR-driven FABP7 isoform produces a chimeric protein with an altered N-terminus, which is essential for cell proliferation and growth in DLBCL cell lines.

Cancer-specific TE-derived chimeric transcripts have also been reported in AML, such as LTR2C-SAGE1 and LTR2B-RHEX transcripts, which are not expressed in healthy myeloid cells [38]. Further underscoring the impact of TE-derived chimeric transcripts, a recent study by Shah and colleagues [39] identified 1088 of such transcripts across nine different blood cancer types. Multiple myeloma exhibited the highest number with 662 events, followed by AML with 159 events, illustrating considerable variation among cancers. Interestingly, genes such as TERT, BUB1, and GABRA3 were frequently involved in chimeric transcript formation. LINEs accounted for 43.57% of these chimeric transcripts, followed by SINEs (29.14%) and LTRs (25.46%).

These findings highlight the diversity and significance of TE-driven chimeric transcripts across blood cancers, with links to disease phenotypes and clinical outcomes. However, ex vivo and in vivo models that directly demonstrate the oncogenic potential of such TE-mediated transcripts remain limited. Functional validation approaches, such as CRISPR/Cas9-mediated deletion or RNA silencing, are necessary to assess the contribution of TE promoters to cellular proliferation and survival. Recent advances in long-read sequencing technologies and computational tools (Box 2) offer greater precision in TE mapping and the identification of these transcripts, potentially revealing more TE-derived transcripts in blood cancers.

Beyond their role in transcriptomic diversity, in some cases TE-derived transcripts are translated into proteins, which may lead to the formation of novel TE-chimeric antigens or cryptic ORFs [39**–**42], that are recognised by immune cells and influence immune responses in haematopoietic malignancies. Aberrant TE expression may also give rise to TE-derived double-stranded RNAs which can activate innate immune sensors such as RIG-I and MDA5, triggering inflammatory pathways. These TE-derived proteins and immunogenic RNAs may contribute to both tumour immunity and immune evasion in haematological cancers, which has been extensively reviewed elsewhere [43] and are not included in this review.

Box 2 Mapping TEs and TE expression Studying TEs presents unique challenges due to their repetitive nature, high copy numbers and variability across individuals. The mappability of TEs decreases with their evolutionary age, as younger TEs tend to have higher copy numbers and fewer mutations, which complicates unique sequence alignment and precise quantification of individual TE copies [145]. To mitigate such issues in mappability, long-read sequencing (LRS) technologies can be deployed to better resolve structural variations in TEs by spanning entire TE regions, thereby reducing alignment ambiguities [146, 147]. LRS also allows the additional identification of single nucleotide polymorphisms (SNPs) and nucleic acid modifications, providing valuable insights into the evolution and epigenetic regulation of individual TEs. These advancements have also facilitated the improvement of reference genomes, such as the completion of the T2T CHM13 genome, which expands the catalogue of human repeats and reveals structural variations previously unresolved in earlier references, such as GRCh38 [148]. Additionally, the development of pangenome references has captured diverse structural variants and complex loci, enabling more accurate TE mapping and analysis across various populations [108, 149].

Studying TE-derived transcripts is a key part of understanding their functions and impacts on the host genomes. For whole RNA transcripts to be sequenced, methodologies like SMART-seq [150] and CELLO-seq [151] have been developed that use a 5′ template-switching oligonucleotide (TSO), which allows the amplification of cDNA from its 5′ end as well as its 3′ end templated by an oligo-dT primer. Both methods are tailored towards single-cell resolution, where the TSOs also contain unique molecular identifier (UMI) sequences to bioinformatically de-convolute the origin of each transcript. In particular, CELLO-seq is tailored towards studying TEs as it incorporates UMIs to uniquely label individual cDNA molecules, enabling bioinformatic correction of PCR and reverse transcription errors. When combined with deep long-read sequencing, this generates high fidelity consensus sequences for each transcript, allowing evolutionarily young TEs to be precisely mapped to their unique loci. Other groups have developed more targeted RNA-sequencing methodologies that enrich for TE-containing transcripts using PCR primers for rapid amplification of cDNA ends, such as 5′-RACE-seq [152], and Cas9-assisted profiling of TEs (CapTE) [153]. Additional techniques such as long-read CAGE-seq [154], TT-seq [155], and PRO-seq [156] target RNA at various points of its life cycle to facilitate the detection of TE-derived transcripts in a non-biased manner. However, these often require greater sequencing depth to allow detection of low-abundance transcripts such as those containing TEs.

While pipeline developments for LRS are still in their infancy, many computational tools have been developed to address issues concerning multi-mapping for short reads. Telescope [157] and TEtranscripts [158] utilise the Expectation-Maximization (EM) algorithm to probabilistically assign multi-mapped reads back to their most likely genomic origins, whereas RepEnrich [159] uses fractional counting methods to resolve ambiguities in read alignment. SQuIRE [160] combines these methods with selective alignment strategies to achieve a high accuracy in quantifying TEs. At the single-cell level, locus-specific resolution has been further enhanced by tools such as SoloTE [161] and IRescue, which combine UMI deduplication with EM algorithms to enhance quantification of single-cell RNA sequencing data [162].

Despite these advancements, many current analyses often do not differentiate between intergenic and intronic/exonic TEs when assessing TE expression. This is problematic, as RNA reads mapping to intronic TEs may result from transcriptional “read-through” events originating from host genes rather than from the TE’s own transcription start site. As a result, apparent TE expression may simply reflect host gene activity, without direct functional relevance for TEs. To interrogate the functional consequences of TE expression, it is crucial to distinguish TEs that actively drive transcription from those that are passively embedded within differentially expressed genes.

### TE-derived lncRNAs

TEs also provide a major promoter source for long noncoding RNAs (lncRNAs). LncRNAs derived from TEs play a crucial role in cancer by modulating gene expression, influencing chromatin structure, and interacting with RNA-binding proteins, as reviewed in [44]. In contrast to protein coding genes, 83% of lncRNAs contain TE sequences with TEs comprising 30–42% of lncRNA sequence lengths [45, 46].

Although much is known about the involvement of lncRNAs in cancer and haematopoiesis, particularly in regulating key processes such as cell proliferation, differentiation, and drug resistance [47], specific regulatory mechanisms linking TEs and lncRNAs in these contexts are less understood and represent an emerging area of research. For instance, lncRNAs such as NALT [48] and UCA1 [49] have been well-documented for their roles in promoting cell proliferation in leukaemia via pathways such as NOTCH and p27^kip1^ suppression, respectively. Similarly, lncRNAs such as HOTAIRM1 [50] and LINC00173 [51] are crucial in regulating haematopoiesis by influencing lineage specification.

However, only a few studies have examined the specific roles of TE-derived lncRNAs in haematopoiesis and blood malignancies. For example, novel lncRNAs identified in B-cell lymphoma are enriched at super-enhancer regions and may utilise TE-derived polyadenylation signals, suggesting a complex regulatory interplay [52]. Additionally, some findings suggest that ERV9 retrotransposon-derived lncRNAs may act in *cis* to stabilize enhancer complexes in erythroid cells, indicating their potential in lineage-specific gene regulation [53]. Yet, the broader roles and mechanisms of TE-derived lncRNAs in blood malignancies remain largely unexplored. The role of TE-derived lncRNAs may remain a ‘hidden’ area: many transcripts annotated as lncRNAs might actually be TE-derived RNAs misannotated as lncRNAs. This represents a significant gap in our understanding, highlighting the need for further research to explore this connection.

### TEs and alternative splicing

Another way that TEs contribute to the diversity of the transcriptome is through alternative splicing, enabling multiple mRNA species to be generated from a single gene. The role of TEs in alternative splicing in the human genome was first demonstrated in Alu elements, where intronic Alu elements generate new exons, thereby affecting expression patterns of numerous genes [54]. Pan-cancer studies have shown that TE-derived alternative splicing events are widespread in cancer [55, 56]. Alu and LINE-1 elements are particularly influential in this process, introducing novel splice acceptor and donor sites, contributing to cancer-specific and recurrent alternative splicing events [55, 56]. Interestingly, the splice donor and acceptor sites within TEs are not randomly distributed but instead cluster at specific hotspots across different TE subfamilies. For example, almost all splicing sites within L1Hs elements are near their 5′ end, whereas all splicing sites within Alus are on their antisense strands [57]. While pan-cancer studies have demonstrated the widespread nature of TE-derived alternative splice sites, these studies predominantly focus on solid tumours and largely exclude haematological cancers, mainly due to the lack of appropriate healthy control tissue. Although the role of TEs in haematological cancers has been less explored, notable examples exist, such as the exonization of an Alu element in the *TIF-IA* gene, which occurs predominantly in leukaemia cell lines and not in healthy cells [58]. Additionally, truncated forms of the *ERBB4* oncogene in anaplastic lymphoma kinase-negative anaplastic large-cell lymphoma (ALK(-)ALCL) have been shown to arise from an endogenous LTR-driven alternative splicing event [59]. The authors reported that two ERBB4 transcript isoforms were generated through the activation of intronic TSSs derived from MLT1H2 and MLT1C elements. Furthermore, a recent comprehensive pan-cancer analysis across 34 cancer types demonstrated that AML samples exhibit among the highest TE-derived exonization events, with an average of 39 events per sample [57]. Moreover, such noncanonical exonization events mediated by TEs can encode tumour-specific immunogenic peptides as demonstrated in lung cancer [60]. Although not yet studied in haematological malignancies, similar immunogenic TE-mediated splicing events may contribute to immune surveillance and hold promise for targeted immunotherapies.

Given the numerous somatic mutations identified in components of the RNA splicing machinery, such as SF3B1, SRSF2, and U2AF1, in haematological malignancies and clonal heamatopoiesis [61], it is crucial to investigate how these mutations may exacerbate TE-derived alternative splicing events, potentially contributing to disease pathology. This highlights a critical need for further research to determine the extent to which TE activity contributes to splicing dysregulation in normal and malignant haematopoiesis, and whether these TE-derived splice variants hold potential as novel therapeutic targets or biomarkers.

### TEs as enhancers

Beyond their roles in generating diverse transcripts, some TEs have been exapted to act as enhancers, influencing gene expression programmes. The first documented example of a TE-derived enhancer in haematopoiesis is the ERV9-LTR element which drives the expression of beta-globin in erythroid cells [62]. This element is conserved during primate evolution and gained enhancer function in embryonic and erythroid cells [63]. In humans, the ERV9 enhancer directly binds to NFY and GATA2 TFs, which subsequently interacts with haematopoietic TFs, facilitating long-range chromatin interactions that enhance beta-globin expression [64, 65]. Interestingly, this enhancer complex is stabilized by ERV9-derived lncRNAs, creating a positive feedback loop that reinforces enhancer-promoter interactions and sustains high levels of beta-globin expression during erythropoiesis [53]. Another TE-derived enhancer influencing normal haematopoiesis is a MIR element, integrated into the genome ~130–160 million years ago during the mammalian/marsupial branchpoint. This ancient MIR element transposed into the enhancer region of the HSC-associated *TAL1* gene, contributing to its regulation in HSCs in humans and mice [66]. Beyond these individual TE examples, genome-wide analyses of the Roadmap Epigenomics Project have shown that TEs contribute to regions with epigenetic enhancer profiles more frequently in haematopoietic lineages than in other human tissues [19]. For instance, 84 TE subfamilies –including ERVs, SINEs, and LINE-2s– are overrepresented in enhancer domains of mouse CD8 + T cells, which are commonly shared with other immune lineages [67]. These immune cell-specific enhancers contain a higher density of TEs compared to active enhancers in non-immune tissues, and TEs are more abundant near immune-related genes. This suggests that TEs have played a critical role in the evolution of immune regulatory networks by integrating functional motifs that fine-tune the expression of immune genes, thereby providing adaptive advantages [67]. Yet, much of the evidence remains correlative. In addition, during the endothelial-to-haematopoietic transition (EHT)—a critical stage in HSC emergence — specific TE subtypes are differentially accessible and overlap with distal enhancer regions, indicating a potential role for TE-derived enhancers in driving HSC formation [68]. However, this study mostly relies on epigenomic signatures that may not directly correlate with functional activity in vivo and the causality of the enhancer roles of TEs in HSCs requires direct functional validation.

Given their contribution to haematopoietic enhancers under physiological conditions, it is plausible that dysregulated TE activity could contribute to the aberrant gene expression seen in haematological malignancies. In fact, genome-wide analyses in various blood cancers have attempted to link epigenetic dysregulations within TEs with their potential enhancer function. For instance, in a study of paediatric acute lymphocytic leukaemia (ALL), TEs, particularly SINEs and LTRs, were frequently found in ALL-specific differentially methylated regions compared to healthy controls. Although this does not provide direct evidence of TE contributions to ALL enhancers, an association was observed between focal hypomethylation in enhancer-marked loci and the abundance of specific TEs [69]. Similarly, another study of 119 chronic lymphocytic leukaemia (CLL) patients from the International Cancer Genome Consortium identified significant differential methylation in retrotransposons, namely LINE-1 and Alu elements [70]. Most of these regions showed hypomethylation with enrichment at enhancers and locus-specific hypomethylation correlated with the differential expression of proximal genes. Additionally, a large cohort study from AML demonstrated that MIR retrotransposons within enhancers of AML-associated genes were highly susceptible to DNA methylation changes in a subtype-specific manner, further linking TE methylation status to their gene regulatory role [71].

While these studies have established correlative links between TE hypomethylation and enhancer activity, direct evidence of TE-derived enhancers shaping gene regulatory networks in haematological cancers is still emerging. For example, Zeng et al. [72] validated the enhancer function of MIR elements, enriched within chromatin accessibility sites in AML patient samples, using a reporter assay in vitro. Furthermore, we previously provided direct evidence for several LTR elements functioning as active enhancers and contributing to transcriptional networks in AML [38]. Accordingly, we identified six LTR subfamilies, including LTR12C and LTR2/2B, that are differentially accessible and bear enhancer-like chromatin signatures in AML patient samples compared to healthy myeloid cells. CRISPR-Cas9 mediated locus-specific genetic deletion and family-level epigenetic silencing of these LTR-derived enhancers led to the downregulation of nearby AML-expressed genes in AML cell lines. Furthermore, we demonstrated that deletion of an LTR2 element within the enhancer region of the *APOC1* gene resulted in significant downregulation of APOC1 expression, reduced cell proliferation, and increased apoptosis, revealing a direct role for TE-derived enhancers in AML cell survival and gene regulatory networks [38]. One limitation of these findings is that they were primarily based on cell line studies, and it remains unclear whether loss of a single enhancer would produce similar effects in primary AML cells, which exhibit greater heterogeneity and epigenetic plasticity. Notably, Grillo et al. further demonstrated that chromatin accessibility at one of these LTR subfamilies, LTR12C, is highly enriched in leukaemia stem cells (LSCs) and is essential for maintaining stemness properties [73]. By applying a CRISPR-dCas9 mediated targeting approach in an AML cell line, they showed that epigenetic silencing of the LTR12C subfamily significantly reduced LSC fractions, confirming that LTR12C accessibility plays a critical role in sustaining LSC identity and hierarchical cellular organisation [73]. These findings highlight how, beyond correlative studies, direct functional assays reveal the enhancer roles of TEs in AML, with implications that may extend to other blood cancers.

### TEs and 3D genome organisation

The human genome is organized into topologically associated domains (TADs), which are regions of DNA anchored at distal sequences to facilitate *cis* interactions within the same domain. Within TADs, *cis*-regulatory elements interact with gene promoters to modulate their expression [74**–**76]. Central to the formation of TADs and chromatin interactions are main architectural proteins and TFs such as CTCF, YY1 and ZNF143 [77, 78].

During haematopoiesis, the 3D structure of the genome undergoes dynamic changes to modulate long-range enhancer-promoter interactions that drive cell-specific transcriptional programmes. CTCF plays a crucial role in regulating these boundaries of higher-order chromatin structures during haematopoiesis [79, 80]. Importantly, CTCF’s role is not limited to normal haematopoiesis; alterations in CTCF binding sites are associated with changes in 3D chromatin conformation in AML [81]. A growing body of evidence suggests that many CTCF binding sites specific to humans and other species are derived from TEs [74, 82, 83]. For instance, Choudhary et al. identified a LINE-1 (L1MC) locus that forms a human-specific chromatin loop mediated by CTCF. Disruption of this LINE-1 element via CRISPR knockout alters gene expression at its interacting locus, directly demonstrating the functional importance of this TE-derived binding site [82]. Notably, TE-derived CTCF binding sites are cell-type specific, with over 80% of these sites specific to leukaemia cell lines compared to lymphoblast cells [84].

YY1 is another looping factor also associated with CTCF binding sites that are better conserved among mammals [85]. YY1 frequently binds to TE-derived sequences, particularly regulatory sequences of LINE-1 elements, with potential implications for transcriptional regulation [86**–**88]. Interestingly, high YY1 expression has been linked to impaired differentiation in AML cells [89]. Similarly, ZNF143 also interacts with CTCF to regulate chromatin organisation [90]. In a mouse model, ZNF143 has been shown to be essential for embryonic HSC development and the formation of a subset of CTCF binding sites [78].

More directly, a recent study in human embryonic stem cells (ESCs) showed that TEs with active histone acetylation marks are significantly enriched at TAD borders and form chromatin loops with genes, suggesting their involvement in chromatin looping and 3D genome organisation [91]. YY1 is enriched at such acetylated LINE-1s, while such LTRs and Alu elements are enriched in the looping factor ZNF143. Moreover, the binding of other key factors such as CTCF and RAD21 were shown to be more pronounced at TEs marked with H4K16ac and H3K27ac, suggesting that histone modifications play a crucial role in TEs’ contribution to 3D chromatin structure via binding to these anchoring and looping factors. Notably, beyond these looping factors, TEs also contribute to chromatin organisation through the formation of insulator elements, independently of CTCF. For example, MIR-derived insulators have been shown to act as chromatin barriers and regulators of gene expression in T cells [92].

Together, these findings highlight the potential of TEs helping to partition the genome into regulatory domains, expanding the functional repertoire of TE-derived elements in 3D genome organisation. These elements may be integral to the dynamic regulation of chromatin organisation in both normal and malignant haematopoiesis. However, direct evidence and functional assays confirming these roles are still lacking. Targeted genetic and epigenetic editing studies are required to definitively establish how these TE-derived sequences modulate chromatin conformation in haematopoietic contexts.

### TE-associated structural variations

Besides their gene regulatory roles, TEs also play a pivotal role in shaping genome structure through their contributions to structural variations. These elements drive genomic changes through various mechanisms including insertional mutagenesis via retrotransposition, generating polymorphisms, and inducing chromosomal rearrangements through recombination of homologous repeats. The following sections explore how each of these processes contributes to genomic instability and disease development, particularly in haematological malignancies.

### Insertional mutagenesis

Although the vast majority of TEs in the human genome are inactive, some young LINE and SINE elements retain their capacity to mobilise, occasionally causing insertional mutagenesis through sporadic retrotransposition events [93]. One of the first described human disorders linked to LINE retrotransposition is haemophilia A, an X-linked blood clotting disorder resulting from a deficiency in Factor VIII [94]. In 1988, Kazazian and colleagues discovered de novo insertions of LINE-1 sequences in exon 14 of the Factor VIII gene in two unrelated patients, providing the first evidence that LINE-1 is an active mobile element in humans. This potential for insertional mutagenesis is particularly relevant in tumours, where epigenetic dysregulations can lead to increased TE activity, although such somatic integrations rarely contribute to oncogenesis. The first report and a unique instance of TE-driven oncogenesis involves a LINE-1 insertion that disrupts the tumour suppressor adenomatous polyposis coli (*APC*) gene, leading to the development of colon cancer [95]. Many studies have since systematically investigated retrotransposition events across various cancer types [96**–**100]. Overall, these events are found in high proportions across many cancers, especially in epithelial tumours (such as lung, prostate, ovarian, and colon cancers). Most of these events occur in intergenic regions or are associated with passenger mutations, underscoring the rarity of TE somatic insertions that drive cancer development and progression. On the other hand, these pan-cancer studies demonstrated that blood cancers show very low to undetectable levels of retrotransposition events [96**–**99]. In line with this, an unexpected tumour suppressive role for LINE-1 elements has recently been described in AML, where LINE-1 silencing is correlated with poor prognosis [101**]**. The authors showed that the loss of MPP8, a component of the HUSH complex, negatively impacts AML progression by promoting the retrotransposition of LINE-1s, which induces a DNA damage response and cell cycle exit. LINE-1 retrotransposition is initiated by the endonuclease activity of ORF2p, which cleaves a single strand of DNA to enable reverse transcription [102]. This process compromises DNA integrity and can lead to the formation of double-strand breaks, either directly or through cellular responses to accumulated DNA damage, potentially activating the DNA damage response and triggering genomic instability [103, 104]. In fact, Gu et al. demonstrated that LINE-1 reactivation in AML increases the formation of γH2A.X foci, a well-established marker for double-strand DNA breaks [101]. This link between high LINE-1 activation and genomic instability in AML may explain why haematological cancers exhibit low LINE-1 activity; clones with high LINE-1 retrotransposition face reduced fitness due to DNA damage, compromising their survival.

### TE-derived polymorphism

While the above mentioned somatic retrotranspositions do not segregate among populations, germline retrotransposition of TEs can result in polymorphic TE loci, where individuals vary in the presence or absence of TE insertions. As such, around 20% of Alu and 15% of LINE-1–mediated deletions are polymorphic in the human population [105]. Recent studies have highlighted the significance of TE insertion polymorphisms, such as ERV-K in the 8q24.13 -8q24.21 region, which is associated with AML cases through disrupting key cancer driver genes such as *MYC* and *PVT1* [106]. Similarly, polymorphic Alu insertions in *MEF2C* and *TAX1BP1* genes have been linked to allele-specific reductions in transcriptional activity in ALL, potentially impacting nearby gene expression and contributing to leukaemogenesis [107].

The pangenomic approach is helpful for such polymorphic TE analyses, because it integrates the wide structural variations across different human populations [108]. Moreover, long-read sequencing technologies enable more precise detection and characterization of polymorphic TEs [109, 110]. Unfortunately, these advanced methodologies have yet to be applied in studies focusing on normal haematopoiesis and blood cancers.

The impact of TEs on genetic diversity is further complemented by single nucleotide polymorphisms (SNPs) within TEs, which can modify nearby gene expression. For instance, an SNP in an LTR5B-derived enhancer element disrupts a MAFK-binding motif, leading to reduced *RPL7L1* expression, a gene that is upregulated in AML [38]. These examples underline the role of both insertion polymorphisms and SNPs within TEs as contributors to genetic and functional diversity in haematological cancers.

### TE-mediated chromosomal rearrangements

TEs can contribute to chromosomal rearrangements through non-allelic homologous recombination and non-homologous repair mechanisms, leading to deletions, duplications, translocations, and inversions in the genome [105]. These TE-mediated chromosomal rearrangements and translocations have been implicated in various cancers, including blood cancers. Particularly, Alu elements act as hotspots for ectopic recombination events due to their high abundance and repetitive nature. These recombination events between non-allelic Alu elements can lead to gene duplications, as in the case of partial tandem duplication of the *MLL* gene frequently occurring in AML patients [111] and tandem duplication of the *MYB* oncogene in T-cell acute lymphoblastic leukemia (T-ALL) [112]. Alu elements have also been reported to be frequently located in close vicinity of breakpoints and implicated in more complex chromosomal rearrangements with various examples predominantly reported in haematological malignancies. Although these events are relatively rare compared to other mechanisms, the presence of Alu elements increases the likelihood of fusion formation, contributing to the pathogenesis of haematological malignancies. These Alu-associated fusions include *MLL-ENL* fusions in ALL [113], Philadelphia chromosome (*BCR-ABL* fusions) in chronic myeloid leukaemia [114] and *MALT1-API2* fusions in mucosa-associated lymphoid tissue (MALT) lymphoma [115]. In addition, LINE-1 elements play a role in promoting large-scale chromosomal rearrangements. Notably, LINE-1 elements account for nearly 19% of chromosomal breakpoints across 17 whole human genomes, implicating them in chromosomal translocations, inversions, and large deletions [116]. Moreover, in Myc-induced mouse models of lymphoma, LINE-1 sequences were frequently found at break sites, with no significant sequence homology, further implicating these elements in driving translocations and other chromosomal rearrangements through erroneous repair mechanisms, similar to Alu-mediated recombination events noted in blood cancers [117]. Though less abundant in the human genome, DNA transposons, such as MER20, also contribute to chromosomal rearrangements. One notable example involves the MER20 transposon, which has been linked to the development of B-cell precursor ALL, with nearly half of the reported translocations in *TCF3-PBX1* ALL involving this transposon [118].

In summary, the involvement of TEs such as Alu, LINE-1, and MER20 in driving chromosomal rearrangements in haematological malignancies suggests their potential role in cancer progression, warranting further investigation into their mechanistic contributions and therapeutic implications.

## Conclusion

TEs have long been considered as genomic bystanders, yet their contributions to both normal and malignant haematopoiesis are increasingly recognised. By reshaping gene-regulatory networks and contributing to structural genome variation, TEs have played a dual role in biology: driving innovation in normal cellular processes and supporting haematopoietic regeneration, while also promoting genomic instability and oncogenesis in haematological malignancies. This review highlights the growing body of research on the importance of TEs in haematopoiesis in both health and disease. In normal haematopoiesis, TEs are instrumental in fine-tuning gene expression programmes, often acting as enhancers or regulatory elements that influence lineage commitment and the functional specialization of haematopoietic cells. In the malignant state, cancer cells can exploit the gene regulatory activities of TEs for their own fitness, promoting oncogene expression. Furthermore, TE-mediated structural variations, such as insertions, deletions, and translocations, are increasingly implicated in the pathogenesis of haematological malignancies, potentially linking their activity to clonal evolution and disease progression.

With advances in experimental and computational approaches, the field is poised to expand our understanding of the multifaceted activities of TEs and their implications in normal and malignant haematopoiesis. Future efforts will focus on establishing causative links of TE activity in these contexts. CRISPR-Cas9 genetic and epigenetic editing offer powerful tools to explore these links by enabling precise manipulation of their activity and allowing researchers to dissect specific TE functions in vitro and in vivo. Along with genome editing tools, future research using long-read sequencing (Boxes 2 and 3) and T2T-CHM13 will allow researchers to map TEs precisely and help to delineate the mechanisms through which TEs influence both normal and pathological processes. The growing recognition of TE-driven processes in haematological malignancies presents an opportunity to develop novel prognostic and therapeutic strategies. Given that TE expression is often cell- and tissue-specific, the activity of oncogenic TEs is typically uniquely activated in tumours, not only making them attractive targets but also potential biomarkers for diagnosis, prognosis and therapy response. From a therapeutic perspective, small-molecule inhibitors or epigenetic drugs that alter TE chromatin states represents a promising approach to modulate TE-driven oncogenic transcriptional programmes. More precisely, the use of lipid nanoparticle-based delivery systems for in vivo epigenetic editing [119] could enable targeted silencing of specific TEs in malignant cells, while RNA-based therapies, including antisense oligonucleotides, may allow selective degradation of TE-derived transcripts. As our understanding of TE-mediated oncogenic processes deepens, integrating these insights into therapeutic strategies has the potential to refine treatment paradigms and improve clinical outcomes in haematological malignancies.

Box 3 Current advances in TE chromatin analysis Given their integral roles in shaping gene regulatory landscapes and serving as *cis*-regulatory hubs, it is essential to precisely analyse chromatin modifications and DNA-protein interactions within TEs. Chromatin immunoprecipitation (ChIP-seq), and Cleavage Under Targets & Release Using Nuclease/Tagmentation (CUT&RUN/Tag) assays are antibody-based approaches used to map histone modifications and DNA-binding proteins onto host genomes, with the latter requiring far fewer input cells compared to traditional ChIP-seq. Assay for Transposase-Accessible Chromatin sequencing (ATAC-seq) is a widely used technique to analyze chromatin accessibility across the genome and to identify potentially active regulatory regions. For TE-centric bioinformatic analyses, computational pipelines have been developed, such as Allo [163], which is based on convolutional neural networks to improve the assignment of multi-mapped reads in ChIP-seq and ATAC-seq data. Experimentally, a number of techniques have been built on the previous principles, resulting in the advent of single-cell and/or long-read chromatin accessibility, histone modification and DNA methylation profiling. Notable examples include scNanoATAC-seq [164] and scNanoSeq-CUT&Tag [165] which profile accessible chromatin and chromatin modifications, respectively, at single cell resolution using long-read nanopore sequencing. NanoHiMe-seq [166] simultaneously profiles DNA methylation and chromatin modifications, and SAM/SMAC-seq [167, 168] profiles chromatin accessibility and modifications coupled with long-read sequencing, thereby increasing the mappability within TEs. In addition, bs-ATLAS-seq was specifically developed to identify non-reference LINE-1 insertions and their locus-specific methylation [169].

Techniques studying the 3D chromatin conformation have also been adapted to long-read sequencing, such as Pore-C [170]. Such chromosome conformation capture methods can then be combined with bioinformatic pipelines to resolve the interacting loci into TADs and chromatin loops. PAtChER is an example of a mapping tool that takes advantage of uniquely mapping HiChIP fragments to improve the mapping of ligated repetitive element sequences based on genomic proximity and therefore improves mapping of locus-specific chromatin modifications [171]. Importantly, loci interacting with TEs can be selected by deploying additional probes complementary to TEs of interest, as has been used with 4Tran-PCR and 4C-Seq [172]. Mediators of these 3D interactions and key regulators of TEs can be specifically validated using techniques like CARGO-BioID [143] and CasID [173] that utilise TE-targeting sgRNAs to direct a fusion of dCas9 and a biotin ligase to biotinylate proteins in the proximity of the target TE sequence. These undergo streptavidin pulled-down and are resolved with liquid chromatography mass-spectrometry (LC-MS) to identify the TE-associated proteomes. These findings can be validated with DiMeLo-Seq, which uses a fusion of an antibody-binding domain and a DNA methyltransferase to direct methylation of adenines on DNA at specific protein-DNA interaction sites, producing N^6^-methyl-deoxyadenosine (m^6^A). These modified residues can then be detected through long-read sequencing to identify the loci of DNA-protein interactions [174].
